# Translation and Validation of the Arabic Version of the Capability Assessment for Diet and Activity (CADA) Questionnaire in Saudi University Employed Women

**DOI:** 10.3390/ijerph18126246

**Published:** 2021-06-09

**Authors:** Jwaher Haji Alhaji, Ghareeb O. Alshuwaier, Nouf Sahal Alharbi, Abdulrahman I. Alaqil, Nora Majed BinSultan, Wadi B. Alonazi

**Affiliations:** 1 Department of Health Sciences, College of Applied Studies and Community Service, King Saud University, Riyadh P.O. BOX 145111 ZIP 4545, Saudi Arabia; 2 Department of Exercise Physiology, College of Sport Sciences and Physical Activity, King Saud University, Riyadh P.O. BOX 145111 ZIP 4545, Saudi Arabia; galshuwaier@KSU.EDU.SA; 3 Department of Health Administration, College of Business Administration, King Saud University, Riyadh P.O. BOX 145111 ZIP 4545, Saudi Arabia; noufsahal@KSU.EDU.SA (N.S.A.); waalonazi@KSU.EDU.SA (W.B.A.); 4 Department of Physical Education, College of Education, King Faisal University, Al-Ahsa P.O. BOX 31982 ZIP 400, Saudi Arabia; aialaqil@kfu.edu.sa; 5 Department of English Language and Translation, College of Languages and Translation, King Saud University, Riyadh P.O. BOX 145111 ZIP 4545, Saudi Arabia; noalsultan@KSU.EDU.SA

**Keywords:** physical activity, Arabic translation, psychometric properties, diet

## Abstract

Background: The Capability Assessment for Diet and Activity (CADA) is a questionnaire that was developed in English and designed to measure the practical barriers and opportunities for diet and physical activity. Objective: This study aimed to translate, culturally adapt, and validate the CADA questionnaire for the Arabic context in a sample of Saudi women employed at a university. Methods: The CADA was translated into Arabic using the forward and backward translation process. The Arabic version was then validated with a sample of 125 female Saudi participants. In order to evaluate the psychometric properties of the Arabic version, Spearman’s rank correlation coefficient was assessed, and a principal component analysis was performed. Results: The translated CADA had good psychometric quality. The content validity analysis revealed a representativeness score of 99.3% and a degree of clarity of 98.6%, indicating excellent compatibility. The principal component analysis showed a single-factor structure. Conclusions: The Arabic version of the CADA questionnaire is now available to assess opportunities to achieve a healthy diet and physical activity level as part of health behavior management, which can lead to more effective interventions for improving people’s health in Arabic-speaking countries.

## 1. Introduction

Non-communicable diseases (NCDs), such as obesity and cardiovascular diseases, as well as their related factors, such as physical inactivity and unhealthy diet, are responsible for the highest mortality and morbidity rates globally [1,2]. Obesity is strongly linked to various health problems, such as type 2 diabetes, cardiovascular disease [1], stroke, high blood pressure, gallbladder disease, osteoporosis, sleep apnea, asthma, and respiratory problems [3]. Evidence also suggests that there are inverse relationships between physical activity and the risk of breast and colon cancer [4]. The results of some studies also imply that physical activity decreases the risk of endometrial and ovarian cancer and may reduce the risk of prostate and lung cancer [5,6].

Measuring physical activity and diet is essential for epidemiological and clinical studies, which are essential to provide accurate data for policymakers, design prevention programs, and promote good health [7]. Moreover, for most instruments that have been approved for measuring diet and physical activity, validity and reliability have been assessed based on mode, frequency, duration, and intensity [8]. Several studies have determined the impact of nutrition (e.g., unhealthy food consumption) and lifestyle (e.g., physical inactivity) on health status (e.g., an increase in the prevalence of obesity and NCDs) in Arabian countries [9,10]. However, the use of valid and reliable tools to measure physical activity and dietary habits in the Arabic language is very limited, especially in Saudi Arabia [11,12]. Additionally, there is growing interest in the role of building knowledge about obesity, most notably in the fields of diet and physical activity [13].

Contemporary research on dietary intake and physical inactivity prevalence in Saudi Arabia has increased significantly, exposing an alarming level of physical inactivity among Saudi adults, especially women [12]. Indeed, data from a national study showed that only 1.9% of Saudi women are physically active, which places most women at high risk of NCDs and mortality [14]. Therefore, the Saudi Arabian government established a national transformation program, Saudi Vision 2030, aiming to enhance the promotion of healthy lifestyles in the Saudi population [15]. Previous studies have also examined the correlations between physical activity, dietary intake, and weight-related conditions [16,17]. In addition, some factors may affect the self-management of environmental and social restrictions on a healthy lifestyle. However, such environmental determinants have not been adequately developed. The framework is a potential approach to address social determinants in practice and planning, as well as evaluating, community response [1,18].

The Capability Assessment for Diet and Activity (CADA) questionnaire was developed by Ferrer et al. [19] to measure practical opportunities for improving physical activity levels and diet quality in adults. It assesses individuals’ perceptions of available resources for healthy diet and activities, and it also examines personal circumstances that may impact people’s ability to access such resources [19]. However, a valid and reliable questionnaire was previously unavailable in the Arabic language for circulation among the Saudi Arabia population [11,12]. Therefore, this study aimed to provide an Arabic translation of the CADA questionnaire and validate it with a sample of Saudi women working as administrators at King Saud University.

## 2. Materials and Methods

### 2.1. Instrument

The CADA questionnaire contains 25 items that can be scored on a 5-point Likert scale ranging from 1 (almost never) to 5 (almost always). The first 14 items of the instrument are dietary items and are categorized into four subscales: diet opportunity (items 1–5), diet barriers (items 6–8), diet knowledge (items 9–11), and diet time (items 12–14). Physical activity is measured by the last eleven items of the CADA questionnaire and is grouped into three subscales: physical activity convenience (items 15–17), neighborhood (items 18–22), and physical activity barriers (items 23–25) [19].

### 2.2. Forward Translation

According to the recommendation of the WHO regarding translating instruments written in English into different languages, the translation of CADA items into Arabic was performed following a forward/backward, multi-step procedure [20]. First, Saudi health academics independently translated the list of items into the Arabic language. Subsequently, the list of items was reviewed and compared with the original instrument, and any disagreements were discussed. This process was conducted in 14 days and resulted in the first draft of the Arabic version of the CADA questionnaire.

### 2.3. Content Validity

The Arabic version of the CADA questionnaire was evaluated to determine whether the language, content, and structure were suitable to assess the relevant indicators among Arabic populations. The validation process was conducted by applying the content validity index (CVI) based on two domains: the representativeness of the domain (R-CVI) and clarity of the domain (C-CVI). The authors assessed both domains for each item and then for the complete scale. An item content validity agreement of 78% or higher indicated an acceptable level of content validity [21,22].

### 2.4. Backward Translation

Following the instrument’s validation, the Arabic version of the CADA questionnaire was translated into English by an independent professional translator who was fluent in both Arabic and English. This translation was performed without reference to the original English CADA instrument (Appendix A).

### 2.5. Validation with Saudi Women

Between December 2019 and January 2020, the current study invited all Saudi women working as administrators at King Saud University to participate. The participants’ body mass index (BMI) was used as a continuous variable; categorization was based on the WHO criteria (BMI: height in meters squared/body weight in kilograms) [18]. All participants were asked to report whether they had any chronic diseases. This study used a convenience sampling method, which resulted in the selection of 125 participants. This number was acceptable as the current study aimed for a sample size five times greater than the number of CADA items [23]. The ethics committee of King Saud University approved the study (reference number: KSU-HE-19-208), and written informed consent was obtained from all the participants.

### 2.6. Statistical Analysis

The statistical analysis procedures involved a descriptive analysis of the CADA items, subscales, and overall scale as well as the demographic characteristics of the participants. Following the same statistical analysis that was applied to the original instrument, the CADA questionnaire scores were calculated as means and standard deviations [19]. Frequencies and percentages were calculated for the participants’ demographic characteristics. Associations between the CADA questionnaire scores and participant characteristics were analyzed using Spearman’s rank correlation coefficient. The inter-item correlation was calculated to evaluate the extent to which items in the scales assessed the same content, and an acceptable level of inter-item correlation ranged between 0.2 and 0.4 [24]. Finally, a principal component analysis was performed to determine the interdependencies among the CADA questionnaire items. The Promax oblique rotation method was used to assess the extraction components. Data analysis was carried out using IBM’s SPSS version 20.0 (IBM Corp, Version 20.0, Armonk, NY, USA). A *p*-value < 0.05 was considered statistically significant.

## 3. Results

A total of 125 subjects participated in this study (40% were between 25 and 60 years old, and 31% were obese). A summary of the participants’ characteristics (including age, BMI, and medical conditions) is presented in Table 1.

Descriptive analysis of the CADA scale scores showed that the mean and SD of the overall CADA score was 3.38 ± 0.50, and the means and SDs of the diet and physical activity scales were 3.53 ± 0.54 and 3.18 ± 0.68, respectively. The CADA subscales ranged from 3.73 ± 0.76 (diet opportunity) to 2.68 ± 1.00 (physical activity barriers), while the individual item scores of the CADA ranged from 4.1 ± 1.06 (item 1: Easy to shop for food) to 2.39 ± 1.25 (item 24: Health limits my activities) (Table 2).

The content validity analysis revealed a representativeness score (R-CVI) of 99.3% and a clarity score (C-CVI) of 98.6%, which indicated excellent agreement. The inter-item correlation analysis showed that there were no significant associations between the mean CADA score and participant characteristics (Table 3).

The principal component analysis with the Promax oblique rotation method indicated that one component had an eigenvalue of 7 and explained 63% of the variation in the CADA questionnaire structure. The feasibility of factor analysis (Bartlett’s test of sphericity) revealed a good value (KMO = 0.67, *p* = 0.001). We then performed EFA with the eigenvalue criteria; the best fit was achieved by a unidimensional structure (Table 2). Furthermore, all factors of the CADA structures had factor loadings of more than 0.4, and 92% of the CADA items had factor loadings were above 0.5.

## 4. Discussion

The current study successfully translated the CADA instrument from English into Arabic. These two languages came from different sources, the Semitic and Indo-European language families; therefore, the researchers used a certified translation process to minimize the linguistic differences related to culture conceptualizations [25,26]. Validation of the psychometric properties of the Arabic version was conducted using a sample of Saudi women. The researchers achieved satisfactory evidence regarding the psychometric properties of the Arabic version of the CADA questionnaire, suggesting that our approach had a high degree of rigor.

The translated CADA questionnaire achieved impressive and satisfactory results, and the responses reflected excellent reliability. In addition, the content validity analysis showed a representativeness score of 99.3% and a degree of clarity score of 98.6%. The average CADA scores were not found to be significantly associated with social or demographic characteristics. This suggests that the translated CADA questionnaire can be used in other Arabian countries while preserving its reliable psychological characteristics [27].

Furthermore, the average overall score of the CADA questionnaire, reflecting the level of healthy diets and physical activity in the current study, was 3.38 out of 5. The mean CADA scores of our results were similar to those of the original instrument, which has been used in studies in the United States, except for the convenience of physical activity scale, for which the results of the current study showed a lower score (3.32 vs. 4.19). This value shows that these Saudi women perceived a lack of nearby places for outdoor physical activity. The results of this study are in accordance with a systematic review by Al-Hazzaa, who explored physical activity barriers among the Saudi population and concluded that there were no appropriate places in which to carry out physical activity, especially for women [12].

Although the benefits of physical activity are well recognized, a high percentage (approximately 98.1%) of the Saudi population, especially women of different ages, are fundamentally physically inactive [28]. Several studies have shown that approximately 78% of adult Saudi women and 78.1% of Saudi teenagers are inactive [29,30]. As a result, physical inactivity in Saudi Arabia presents a public health burden. This study also confirmed that about 43% of the participants were overweight (BMI between 25.0 and 30) and 31% were obese (BMI ≥ 30), in agreement with previous studies that indicated high BMI in women in Arabic countries in general [31,32].

The inconvenience of practicing physical activity among Saudi women may be related to environmental, individual, and organizational barriers [33]. The high level of physical and social barriers and the hot weather discourages walking and exertion outdoors, and an unfriendly built environment hinders exercise and promotes a car-dependent culture. These factors contribute to devaluing and discouraging exercise [34,35]. Moreover, at the community level, previous studies indicated that cultures and norms are viewed as a barrier to physical activity among Arabian women [36]. For example, many women wear traditional clothing in public (e.g., the abaya, which is “a traditional loose-fitting outer garment that is worn by many women in parts of the Islamic world”), which may make it difficult for them to participate in outdoor physical activity [37,38]. On the other hand, at the policy level, barriers are related to the lack of allocation of funding for sports, especially for women. In a study conducted in Saudi Arabia, participants reported that there was limited funding for women to join sports clubs, and gym memberships were typically expensive [39].

Effectively responding to the epidemics of obesity and chronic disease, especially in developing countries, requires intervention models that account for important drivers of diet and physical activity patterns [40]. The authors believe that the CADA questionnaire is a vital tool for understanding the relationships between different lifestyle factors and developing effective promotional programs for healthy eating and increased physical activity [41]. Public health authorities continue their efforts to raise awareness about the importance of physical activity and a healthy lifestyle [13]. With the Quality of Life Program, one of the Saudi Vision 2030 ambitions, the Saudi Arabian government aims to enhance healthy lifestyles among the population [15]. Therefore, the Arabic version of the CADA can contribute to assessing barriers and opportunities for physical activity and healthy diet not only in Saudi Arabia but also in other Arabic-speaking countries in the Middle East and North Africa, where the questionnaire can be culturally adapted to the studied population [42]. Thus, the conclusions in this study carry important implications for public health policies and intervention programs in these regions.

## 5. Conclusions

This research created an Arabic version of the CADA questionnaire, which the researchers believe to be an important measure for policymakers in the Arab region as a guide for improving the quality of life of women. To our knowledge, this study is the first to translate and cross-culturally validate the CADA into Arabic. One limitation of this study was that the sample only comprised women who shared the same employment environment and had similar jobs. However, although the sample in the study was not representative of the general Saudi population, it covered a wide range of ages and educational statuses and included individuals from different regions of Riyadh who worked at King Saud University. Further evaluation of the questionnaire is needed with other genders, age groups, and occupations, as well as in different populations of other Arabic-speaking countries.

## Figures and Tables

**Table 1:** Characteristics of the study sample (*n* = 125).

Age (Years)	*n* (%)
25–35	24 (19.2)
36–40	49 (39.2)
41–50	40 (32)
>50	12 (9.6)
BMI (kg/m^2^)	
Normal: 18.6–24.9	32 (25.6)
Overweight: 25–29.9	54 (43.2)
Obese: ≤30	39 (31.2)
Mean ± SD	28.04 ± 4.72
Medical conditions	
Hypertension	9 (7.2)
Diabetes	8 (6.4)
High cholesterol	7 (5.6)
Hypothyroidism	7 (5.6)
Asthma	4 (3.2)
Depression	1 (0.8)
Gestational Disease	1 (0.8)
Degenerative Joint Disease	1 (0.8)
Renal Disease	1 (0.8)

BMI: body mass index; SD: standard deviation.

**Table 2:** Descriptive of CADA items and scale characteristics (*n* = 125).

Scales/Items	Mean/SD	95% CI	Item–Scale Correlation ^a^	Item–Total Correlation ^b^
Diet	3.53 (0.54)	(3.43, 3.63)		
Diet opportunity	3.73 (0.76)	(3.59, 3.86)		
Easy to shop for food	4.10 (1.06)	(3.91, 4.29)	0.67	0.46
Can afford fresh fruit and vegetables	4.00 (1.11)	(3.80, 4.19)	0.74	0.50
Can afford lean meat or fish	3.57 (1.17)	(3.36, 3.78)	0.75	0.48
High-quality fruit and vegetables	3.46 (1.20)	(3.25, 3.76)	0.69	0.46
Too expensive to buy groceries over the entire month	3.52 (1.33)	(3.28, 3.75)	0.46	0.26
Diet barriers	3.11 (0.83)	(2.96, 3.25)		
Illness gets in the way of cooking meals	3.63 (1.20)	(3.41, 3.84)	0.64	0.35
Too tired to cook my own meals	3.07 (1.27)	(2.84, 3.29)	0.71	0.23
Feeling depressed keeps me from food shopping	2.63 (1.18)	(2.42, 2.84)	0.70	0.18
Diet knowledge	3.66 (0.95)	(3.50, 3.83)		
Know how to eat healthy foods	3.86 (1.10)	(3.66, 4.05)	0.82	0.43
Know how to choose healthy meals at restaurants	3.53 (1.20)	(3.32, 3.74)	0.91	0.51
Know where to shop for healthy food	3.60 (1.09)	(3.41, 3.80)	0.79	0.59
Diet time	3.48 (0.85)	(3.33, 3.63)		
Taking care of family leaves little time for cooking	3.39 (1.21)	(3.17, 3.60)	0.67	0.44
Schedule leaves little time for food shopping	3.51 (1.16)	(3.30, 3.71)	0.77	0.26
Schedule gives me little time for cooking	3.55 (1.13)	(3.35, 3.75)	0.75	0.29
Physical Activity	3.18 (0.68)	(3.06, 3.30)		
Physical activity convenience	3.20 (1.22)	(2.98, 3.42)		
Places nearby for outdoor physical activity	3.32(1.46)	(3.06, 3.57)	0.84	0.68
Places are open when I want indoor activity	3.25 (1.48)	(2.99, 3.51)	0.91	0.61
Can afford to join a gym	3.04 (1.43)	(2.78, 3.29)	0.75	0.37
Neighborhood	3.47 (0.89)	(3.31, 3.63)		
Easy to walk to places in my neighborhood	3.36 (1.45)	(3.11, 3.65)	0.78	0.56
Places where I can be active without needing to pay	3.39 (1.34)	(3.15, 3.62)	0.39	0.08
Often see people walking in my neighborhood	3.44 (1.31)	(3.21, 3,68)	0.79	0.59
People generally feel safe in my neighborhood	3.80 (1.19)	(3.58, 4.01)	0.67	0.59
Neighborhood is well-lit for evening activities	3.36 (1.38)	(3.12, 3.61)	0.71	0.49
Physical activity barriers	2.68 (1.00)	(2.50, 2.86)		
Illness gets into the way of being active	2.72 (1.29)	(2.49, 2.95)	0.88	0.21
Health limits my activities	2.39 (1.25)	(2.16, 2.61)	0.82	0.09
Feeling depressed keeps me from being physically active	2.94 (1.27)	(2.71, 3.17)	0.67	0.21
Overall CADA Score	3.38 (0.50)	(3.29, 3.47)		

SD: standard deviation; CI: confidence interval. ^a^ Correlation between item and corresponding scale. ^b^ Correlation between item and overall CADA score.

**Table 3:** Correlation between participant characteristics and CADA scores.

Demographic Characteristics	CADA Score	
	R	*p*-Value
Age	0.07	0.92
BMI	0.01	0.11
Medical conditions	−0.05	0.93

## Data Availability

Not applicable.

## Appendix A

**Table A1:** The Arabic Version of the Capability Assessment for Diet and Activity (CADA) Questionnaire.

العنصر		العامل الأولي	العامل
سهولة شراء وتسوق الطعام	.1	فرص الحمية المتاحة	مصادر الحمية الغذائية
القدرة على التزود بالخضروات والفواكه الطازجة	.2		
. القدرة على الحصول على اللحوم الخالية من الدهون أو الأسماك	.3		
.الخضروات والفواكه عالية الجودة	.4		
.التكلفة المادية عالية جداً لشراء المواد الغذائية على مدى شهر	.5		
تأتي الامراض بسبب طريقة طهي الطعام	.6	معوقات اتباع الحمية الغذائية	عوامل ترك الحمية الغذائية
.أحس بالإرهاق عندما أقوم بطهو طعامي بنفسي	.7		
يمنعني الإحباط من التسوق للطعام	.8		
أعرف كيف أتناول الأطعمة الصحية	.9	المعرفة بالحمية الغذائية	
.أعرف كيف أختار الاطباق الصحية في المطاعم	.10		
.أعرف أماكن التسوق الخاصة بالأطعمة الصحية	.11		
.الاعتناء بالعائلة يمنعني أن اقضي الوقت في الطبخ	.12	وقت الحمية الغذائية	
.جدولي اليومي يسمح لي بقضاء وقت قليل من أجل التسوق للطعام	.13		
جدولي اليومي يسمح لي بقضاء وقت قليل في المطبخ	.14		
.أختار الأماكن القريبة من أجل اداء النشاطات البدنية الخارجية	.15	سهولة ممارسة النشاط البدني	مصادر النشاط البدني
.أختار أماكن واسعة في المنزل لممارسة النشاط البدني	.16		
يمكنني تحمل تكلفة الاشتراك في نادي رياضي	.17		
.توفر مواقع للمشي في الحي السكني	.18	مدى ملاءمة الأماكن المجاورة لممارسة النشاط البدني	
.بعض الأماكن تمكنني من أداء النشاطات الرياضية دون الحاجة إلى دفع المال	.19		
.كثيراً من الأشخاص في حينا يشاركونني المشي في أغلب الأوقات	.20		
.بشكل عام، يشعر الناس غالباً بالأمان في حينا	.21		
توجد في الحي إنارة تعمل ليلاً لممارسة الانشطة الرياضية	.22		
.حالتي الصحية تعيق من أداء الأنشطة البدنية	.23	عوائق ممارسة النشاط البدني	عوامل تحولات النشاط البدني
.صحتي تمنعني أن امارس نشاطاتي	.24		
.الشعور بالإحباط يمنعني من أن أكون نشيطاً بدنياً	.25		
